# Magnetic resonance imaging characteristics in patients with spondyloarthritis and clinical diagnosis of heel enthesitis: post hoc analysis from the phase 3 ACHILLES trial

**DOI:** 10.1186/s13075-022-02797-8

**Published:** 2022-05-16

**Authors:** X. Baraliakos, P. Sewerin, E. de Miguel, E. Pournara, C. Kleinmond, A. Shekhawat, C. Jentzsch, A. Wiedon, F. Behrens, on behalf of the ACHILLES study group

**Affiliations:** 1 Rheumazentrum Ruhrgebiet-Ruhr-University Bochum, Herne, Germany; 2 Hiller Research Unit, University Hospital Duesseldorf, Duesseldorf, Germany; 3 Rheumatology Department, Hospital Universitario La Paz, Madrid, Spain; 4 Novartis Pharma AG, Basel, Switzerland; 5 ClinProject GmbH, Eurasburg, Germany; 6 Novartis Healthcare Private Limited, Hyderabad, India; 7 Novartis Pharma GmbH, Nürnberg, Germany; 8 CIRI/Rheumatology and Fraunhofer TMP, Goethe-University, Frankfurt, Germany

**Keywords:** Spondyloarthritis, Inflammation, Structural damage, Heel, Enthesitis, HEMRIS, MRI scoring system

## Abstract

**Objective:**

To investigate the imaging characteristics and clinically assess heel enthesitis in spondyloarthritis (SpA) by applying in a post hoc analysis the Heel Enthesitis Magnetic Resonance Imaging Scoring system (HEMRIS) in blinded and centrally-read MRI data from the ACHILLES trial (NCT02771210).

**Methods:**

ACHILLES included patients (≥18 years) with active psoriatic arthritis or axial SpA with clinical and MRI-positive heel enthesitis refractory to standard treatment. Patients were randomized to receive subcutaneous secukinumab 150/300 mg or placebo. At week 24, patients on placebo were switched to secukinumab treatment. MRI-positive heel enthesitis was confirmed in all patients by local investigators. MRIs were performed at 3 timepoints: screening and weeks 24 and 52. In the present analysis, all MRIs were re-evaluated by 2 blinded central readers in a consensus read fashion for a priori defined MRI parameters based on HEMRIS.

**Results:**

At screening, 171/204 (83.8%) of patients presented with entheseal inflammation and/or structural damage, considering both the Achilles tendon and plantar fascia. Pathologies were more evident in the Achilles tendon area compared to the plantar aponeurosis. The most frequent pathologies were intra-tendon hypersignal and retrocalcaneal bursitis. The mean total entheseal inflammation score at screening in the Achilles tendon area was 2.99 (*N*=204) and the mean change (standard deviation [SD]) from screening to weeks 24 and 52 was − 0.91 (1.99) and − 0.83 (2.12) in the secukinumab group vs − 0.48 (1.86) and − 0.80 (1.98) in the placebo-secukinumab group, respectively. The mean total structural damage score at screening was 1.36 (*N*=204) and the mean change (SD) from screening to weeks 24 and 52 was 0.00 (0.65) and − 0.06 (0.56) in the secukinumab group vs 0.08 (0.48) and 0.04 (0.75) in the placebo-secukinumab group, respectively.

**Conclusions:**

Based on the newly developed HEMRIS, entheseal inflammation and/or structural damage was confirmed in 83.3% of ACHILLES patients. Pathologies were more evident in the Achilles tendon area compared to plantar fascia, with the inflammatory parameters being more responsive with secukinumab treatment compared to placebo. The present analysis, with detailed information on individual MRI parameters, contributes to the scientific debate on heel enthesitis.

**Trial registration:**

ClinicalTrials.gov NCT02771210.

**Supplementary Information:**

The online version contains supplementary material available at 10.1186/s13075-022-02797-8.

## Introduction

Enthesitis is a hallmark feature of spondyloarthritis (SpA), including psoriatic arthritis (PsA) and axial SpA (axSpA), and is proposed as the primary lesion in spondyloarthropathies [1–3]. However, little is known regarding differences of enthesitis within the spectrum of SpA. Entheses of the lower extremities is involved more frequently than those of the upper limbs. Although not systematically evaluated, the heel (Achilles tendon and plantar fascia insertions) is reported to be affected most frequently [4]. Entheseal pain can be severe, disabling, and continuous, and can last for several years [5]. Enthesitis in patients with axSpA has been associated with worse disease activity and quality of life than those with no enthesitis [6].

Furthermore, diagnosis of enthesitis still presents a challenge, irrespective of assessment by clinical or imaging criteria. Clinically, enthesitis is usually assessed using scores such as the Leeds Enthesitis Index (LEI), Maastricht Ankylosing Spondylitis Enthesitis Score (MASES), or Spondyloarthritis Research Consortium of Canada (SPARCC) with variable correlation with measures of disease activity and function [7]. These indices assess pain and tenderness in entheseal sites in a binary fashion and hence are more a measure of “pain” rather than a measure of “inflammation.” Moreover, they are influenced by the variable intensity of pressure and individual pain perception.

Recent advances in imaging including magnetic resonance imaging (MRI) and ultrasound (US)/power Doppler ultrasound (PDUS) allow for the evaluation of the extent of inflammation at the entheseal site and have evolved as important tools for the diagnosis and monitoring of enthesitis [8, 9]. Both US and MRI can reveal soft-tissue inflammation, although only MRI can depict inflammatory areas in bone [10]. This is a unique advantage of identifying peri-entheseal inflammation with adjacent bone marrow edema (BME), potentially facilitating early diagnosis in SpA [11]. However, the diagnosis of enthesitis with imaging can be challenging given the low vascular nature of entheses at bony attachment sites and the low density of vessels in surrounding ligaments and tendons. The Outcome Measures in Rheumatology (OMERACT) group have developed a scoring system for the hand (Psoriatic Arthritis MRI Scoring system [PsAMRIS]) and most recently for the heel (Heel Enthesitis MRI Scoring system [HEMRIS]) to allow for comparisons of clinical study results [12–14]. Despite these advances, the diagnosis and monitoring of enthesitis remains a challenge due to the limited number of prospective clinical studies and the potential divergence between pain at entheseal sites and the objective signs of inflammation.

ACHILLES (NCT02771210) is the largest prospective placebo-controlled randomized controlled trial so far, investigating both clinical and imaging endpoints, with blinded and centrally-read MRI data on heel enthesitis in patients with SpA [15]. ACHILLES is focusing on heel enthesitis, investigating not only clinical endpoints but also blinded and centrally-read MRI data of the heel. A *post hoc* analysis applying HEMRIS in blinded and centrally read MRI data of the heel from the ACHILLES trial (*N*=204) is reported here.

## Methods

### Study design

ACHILLES is a 2-treatment arm, randomized, parallel-group, double-blind, placebo-controlled study in patients with PsA or axSpA [15]. Patients were randomized to receive subcutaneous (s.c.) secukinumab 150 mg, 300 mg or placebo at baseline, weeks 1, 2, 3, and 4, followed by once every 4 weeks. At week 24, patients on placebo were switched to secukinumab 150 or 300 mg.

### Patients

Patients (≥18 years) with active SpA and clinical heel enthesitis confirmed by imaging were eligible to participate in the trial. Active SpA was defined as either axSpA with Bath Ankylosing Spondylitis Disease Activity Index (BASDAI) ≥4 (0-10) at baseline or PsA fulfilling the CASPAR (ClASsification for Psoriatic ARthritis) criteria with symptoms for at least 6 months and ≥1 tender joint out of 78 and ≥1 swollen joint out of 76 at baseline. Clinical heel enthesitis was defined as swelling and tenderness at the insertional site of the Achilles tendon into the calcaneus (binary pain assessment). Heel enthesitis must have been refractory to standard treatment (either non-steroidal anti-inflammatory drugs or tumor necrosis factor-inhibitors) with an onset of heel pain ≥1 month prior to baseline.

The study was carried out in accordance with the principles of the Declaration of Helsinki, International Conference of Harmonisation Good Clinical Practice guidelines, and all applicable laws and regulations, with written informed consent obtained from all enrolled patients.

### Image acquisition

The MRI protocol of the study consisted of three mandatory sequences: T1-weighted Turbo Spin-Echo, T1-weighted Fast Spin-Echo, and T2-weighted short inversion time inversion-recovery [STIR] and predefined sequence parameters (details as reported previously) [16]. MRIs were performed at 3 timepoints: screening, week 24, and week 52. No preparative drugs, contrast agents, or radionuclide agents were used. If both feet were affected, the foot with the highest pain level according to the patient’s decision was examined.

### Local assessment

Heel enthesitis by MRI was assessed at screening by either the local radiologist or rheumatologist at the study site to determine the eligibility of the patient; the local rheumatologist was allowed to overrule the local radiologist’s assessment with respect to the positivity of the MR images. The MRI was positive for heel enthesitis if tendinitis with/without bursitis and/or BME with/without concomitant erosions in the insertional area of the Achilles tendon and/or the plantar aponeurosis was present. Based on the screening image, the investigator provided confirmation of whether a patient fulfills the inclusion criterion of MRI-positive heel enthesitis (yes/no) without a detailed evaluation of MRI parameters.

### Central reading

After inclusion in the study and independent of local assessment, the MR images were evaluated by two central readers in a consensus-read fashion. The readers were blinded for patient identification, site, timepoint, treatment, and clinical assessment and were experienced professionals with scientific and technical expertise in imaging modalities. Details of the central reading assessments have been reported previously [16]. The initial MRI evaluation was performed based on PsAMRIS adapted to the heel with outcomes reported in the primary manuscript [15]. The present report presents a *post hoc* analysis based on the re-evaluation of all MRIs by applying the recently developed HEMRIS.

### HEMRIS

HEMRIS was developed by OMERACT to assess enthesitis of the heel in patients with SpA [13]. Inflammatory and structural parameters are each scored on a semi-quantitative scale of 0–3 (none to severe; detailed definitions for each severity grade can be found in the atlas of the OMERACT HEMRIS) [17]. The total entheseal inflammation score is the sum of all inflammatory parameter scores in the area of the Achilles tendon, namely intra-tendon hypersignal, peri-tendon hypersignal, retrocalcaneal bursitis, and BME, thus ranging from 0 to 12. Similarly, the total entheseal inflammation score in the area of the plantar fascia is the sum of intra-aponeurosis hypersignal, peri-aponeurosis hypersignal, and BME (without bursitis), ranging from 0 to 9. The total structural damage score is the sum of all structural parameters scores, namely tendon thickening/aponeurosis thickening, bone spur, and bone erosion, ranging from 0 to 9 in each of the locations.

### Statistical analysis

Categorical variables (qualitative and quantitative imaging parameters) were presented as descriptive summary statistics. Summaries included relative and absolute frequencies for each category. Continuous variables (MRI scores) were presented as mean with standard deviation (SD). From baseline to week 24, MRI data were missing for 14 patients due to study discontinuation, for 5 subjects although they completed week 24, and for 4 patients the MRIs were excluded from the analysis as they were assessed outside the visit window. From baseline to week 52, additional MRIs of 31 patients were missing due to study discontinuation, missed assessment, or were outside the visit window.

A chi-square analysis was performed to investigate potential associations of the inflammatory imaging parameters in the area of the Achilles tendon (intra-tendon hypersignal, peri-tendon hypersignal, bursitis, and BME) with clinical parameters directly related to the heel (Achilles tendon enthesitis as assessed by LEI, heel pain, and heel enthesopathy, as reported by patients). Changes in imaging and clinical parameter from screening to week 24 were categorized as either “improvement” or “no-improvement” to calculate *P* values.

## Results

Overall, 204 (128 PsA and 76 axSpA) SpA patients with heel enthesitis were randomized in the ACHILLES trial. At baseline, the mean (SD) age of the patients randomized to the secukinumab group was 47.8 (11.3) years and those randomized to the placebo group were 47.7 (11.0) years, with a mean (SD) body mass index of 29.0 (6.3) and 29.7 (6.3), respectively. Patients in the secukinumab and placebo groups reported a mean (SD) heel pain of 6.4 (2.3) and 6.2 (2.1) on a 0 to 10 numerical rating scale. Of the PsA patients, the mean (SD) time (months) since the onset of enthesitis was similar in both secukinumab (33.9 [51.8]) and placebo (33.7 [62.2]) groups, whereas in axSpA patients, it was 39.3 (73.0) and 28.9 (51.9) in the secukinumab and placebo groups, respectively [15].

The post hoc analysis of the screening MR images (based on HEMRIS) revealed that 84% (171/204) of these patients presented with entheseal inflammation and/or structural damage of the heel, considering both the areas of the Achilles tendon and plantar aponeurosis. Inflammatory and structural changes were identified for 76% (156/204) and 64% (131/204) of the patients, respectively.

**Fig. 1:**
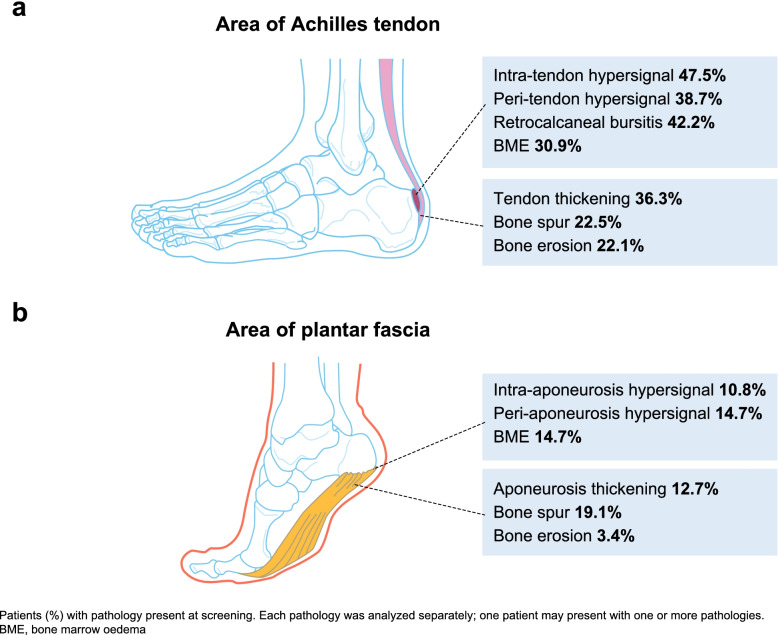
Qualitative assessment at screening based on HEMRIS. **A** Area of Achilles tendon. **B** Area of plantar fascia. Patients (%) with pathology present at screening. Each pathology was analyzed separately; one patient may present with one or more pathologies. BME, bone marrow edema

**Fig. 2:**
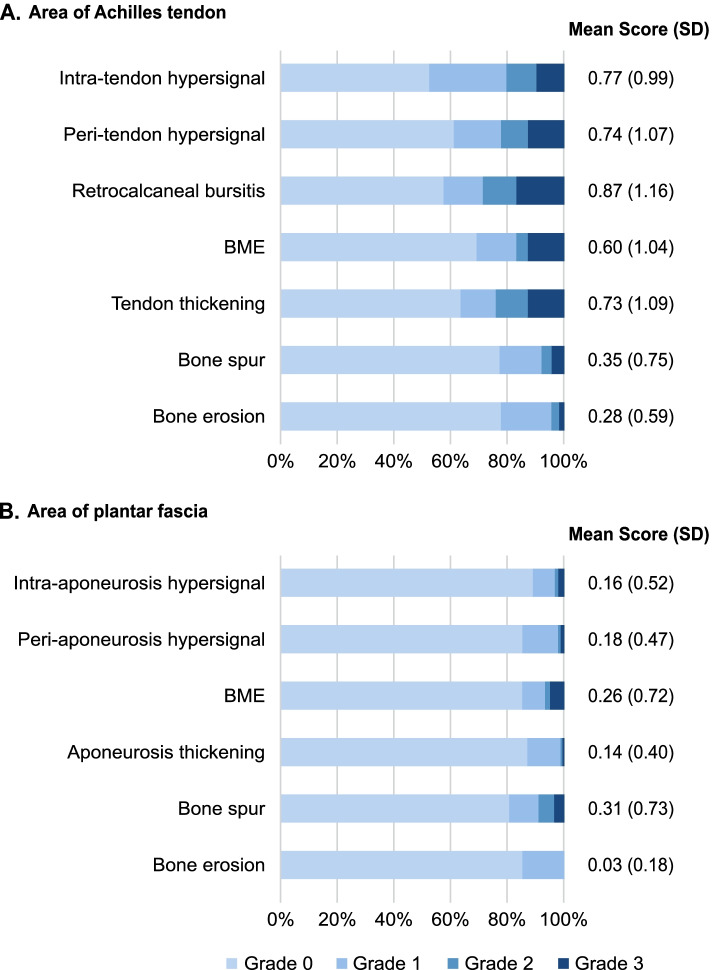
Quantitative assessment at screening based on HEMRIS. **A** Area of Achilles tendon. **B** Area of plantar fascia. Each pathology scored from 0 (not present) to 3 (severe/large). BME, bone marrow edema; SD, standard deviation

As depicted in Fig. 1, pathologies were more frequent in the area of the Achilles tendon compared to the area of plantar aponeurosis. The most frequent pathologies were intra-tendon hypersignal and retrocalcaneal bursitis which were found in 47.5% and 42.2% of all patients, respectively. The quantitative assessment at screening, based on HEMRIS describing MRI pathologies by severity grade 0 (not present) to 3 (severe), is shown in Fig. 2. The mean severity grades were generally higher in the area of the Achilles tendon, ranging from 0.28 (bone erosion) to 0.87 (bursitis) as compared to the area of plantar aponeurosis, with mean severity grades ranging from 0.03 (bone erosion) to 0.31 (bone spur). The specific inflammatory and structural parameters for both the Achilles tendon and plantar fascia were not present in majority of patients. The highest severity grade was most often scored for retrocalcaneal bursitis, with 16.7% of patients presenting with bursitis grade 3 at screening (i.e., maximal diameter of hypersignal in the shorter of two perpendicular dimensions ≥1.0 cm).

In terms of quantitative assessment, the mean (SD) of the total entheseal inflammation and the total structural damage score in the area of the Achilles tendon was 2.99 (3.27) and 1.36 (1.68), while in the area of plantar fascia was 0.59 (1.33) and 0.49 (0.91), respectively (Table 1).

**Table 1:** Total entheseal inflammation score and total structural damage score at screening

	SEC (***N***=102)	PBO (***N***=102)	All patients (***N***=204)
**Area of Achilles tendon**			
**Total entheseal inflammation score (0–12)**			
Mean (SD)	3.04 (3.12)	2.93 (3.42)	2.99 (3.27)
Median	2.00	1.00	2.00
Min, Max	0.00, 12.00	0.00, 12.00	0.00, 12.00
**Total structural damage score (0–9)**			
Mean (SD)	1.45 (1.84)	1.26 (1.51)	1.36 (1.68)
Median	1.00	1.00	1.00
Min, Max	0.00, 8.00	0.00, 6.00	0.00, 8.00
**Area of plantar fascia**			
**Total entheseal inflammation score (0–9)**			
Mean (SD)	0.55 (1.13)	0.64 (1.51)	0.59 (1.33)
Median	0.00	0.00	0.00
Min, Max	0.00, 6.00	0.00, 8.00	0.00, 8.00
**Total structural damage score (0–9)**			
Mean (SD)	0.47 (0.90)	0.51 (0.92)	0.49 (0.91)
Median	0.00	0.00	0.00
Min, Max	0.00, 5.00	0.00, 3.00	0.00, 5.00

*N* total number of patients, *PBO* Placebo, *SD* Standard deviation, *SEC* Secukinumab

### Assessments at weeks 24 and 52

**Fig. 3:**
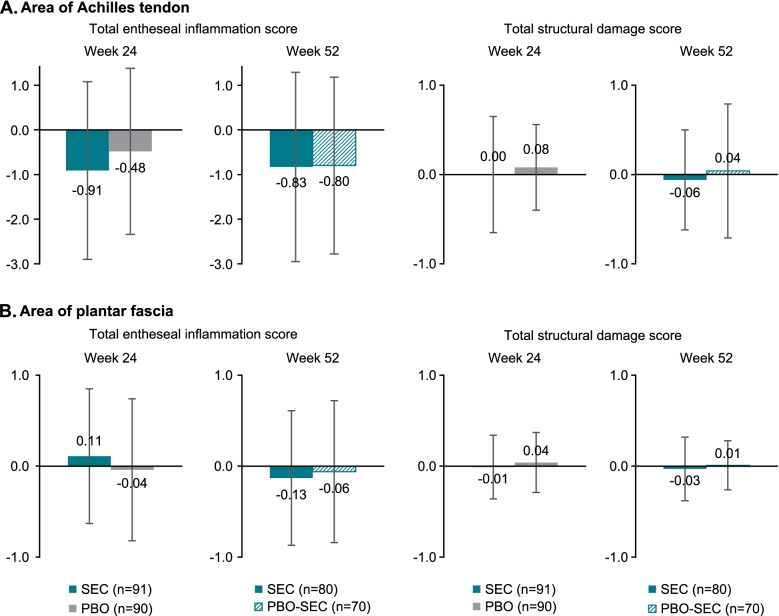
Change in total entheseal inflammation score and total structural damage at weeks 24 and 52. **A** Area of Achilles tendon. **B** Area of plantar fascia. Patients on PBO received active SEC treatment from week 24 to week 52. *n*, number of patients; PBO, placebo; SEC, secukinumab

In the area of the Achilles tendon, the mean (SD) decrease from screening to week 24 in the total entheseal inflammation score was higher in patients treated with secukinumab compared to placebo, − 0.91 (1.99) vs − 0.48 (1.86), respectively (Fig. 3). In the area of plantar aponeurosis, the mean changes (SD) from screening to week 24 in total entheseal inflammation score were low in both the secukinumab and the placebo group, 0.11 (0.74) and − 0.04 (0.78), respectively. The mean change over time in total structural damage score was minimal in both areas.

Further analysis in the area of the Achilles tendon showed a mean (SD) decrease to week 24 in total entheseal inflammation score of − 1.16 (2.20) and − 0.50 (1.52) in the secukinumab group and − 0.60 (1.84) and − 0.27 (1.89) in the placebo group for PsA and axSpA patients, respectively (Supplementary Fig. 1).

**Fig. 4:**
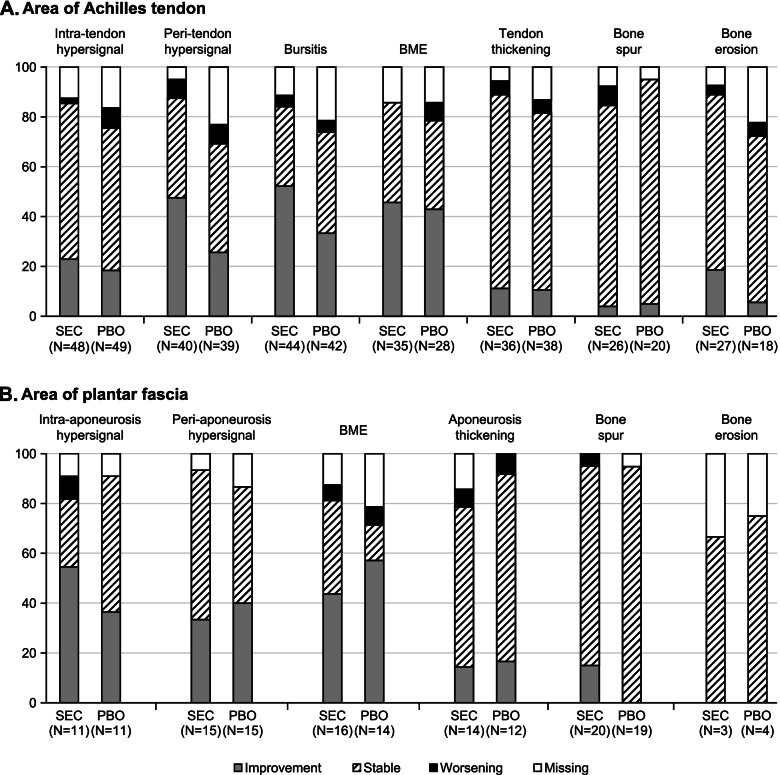
Changes in individual MRI parameter at week 24: Proportion of subjects with improvement/worsening of MRI parameter. **A** Area of Achilles tendon. **B** Area of plantar fascia. MRI, magnetic resonance imaging; *N*, total number of patients; PBO, placebo; SEC, secukinumab

Figure 4 displays the proportion of patients with improvement/worsening of any pathology present at screening up to Week 24. For the area of Achilles tendon (Fig. 4A), an improvement in all inflammatory parameters was observed for a higher proportion of secukinumab treated patients compared to placebo. Retrocalcaneal bursitis and intra-tendon hypersignal improved for 52.3% and 22.9% of the patients in the secukinumab group and 33.3% and 18.4% in the placebo group, respectively. The structural parameters (tendon thickening, bone spur, bone erosion) at the Achilles tendon remained stable up to week 24 in the majority of patients in both treatment groups. For the area of plantar aponeurosis, an overview of the proportion of patients with improvement/worsening of individual pathologies from screening to week 24 is presented in Fig. 4B; only a few patients presented with MRI findings in this region at screening compared to the area of Achilles tendon. Furthermore, Supplementary Table 1 provides an overview of subjects with newly developed MRI parameters over time. Overall, very few patients were found with newly developed pathologies from screening to week 24. However, 7 patients out of 60 in the placebo group and 1 out of 58 in the secukinumab group developed retrocalcaneal bursitis.

The changes from screening to weeks 24 and 52 in specific MRI parameters based on HEMRIS mean scores in the overall ACHILLES population (including patients that did not present with the respective parameter at screening) are presented in Supplementary Table 2.

Changes in imaging and clinical parameters from screening to week 24 were investigated for the potential association. As shown in Table 2, an association was revealed for peri-tendon hypersignal and heel enthesopathy (*P*=0.003) and retrocalcaneal bursitis and heel pain (*P*=0.021) while no association to a clinical parameter was found for intra-tendon hypersignal and BME.

**Table 2:** Association of clinical parameter and imaging parameter (inflammatory parameter in the area of Achilles tendon) assessed with categories improvement/no improvement from screening to week 24

Parameter	Achilles tendon enthesitis	Heel pain	Heel enthesopathy
**Intra-tendon hypersignal**	*P*=0.496 (*n*=83)	*P*=0.903 (*n*=74)	*P*=0.724 (*n*=74)
**Peri-tendon hypersignal**	*P*=0.335 (*n*=68)	*P*=0.773 (*n*=58)	*P*=0.003 (*n*=58)
**Bursitis**	*P*=0.092 (*n*=72)	*P*=0.021 (*n*=61)	*P*=0.095 (*n*=61)
**BME**	*P*=0.057 (*n*=54)	*P*=0.303 (*n*=46)	*P*=0.268 (*n*=46)

Achilles tendon enthesitis, single tender point out of the 6-point LEI (binary assessment 0,1); heel pain, 0–10 NRS reported by patients; heel enthesopathy, 0–100 VAS reported by patients; Associations calculated based on chi-square test with categories improvement/no-improvement (parameters that remained stable from screening to Week 24 were considered as “no-improvement”); all subjects with respective HEMRIS parameter present at screening were considered for the analysis

*BME* Bone marrow edema, *HEMRIS * Heel Enthesitis Magnetic Resonance Imaging Scoring system, *LEI* Leeds Enthesitis Index, *NRS* Numeric rating scale, *VAS* Visual analog scale

## Discussion

ACHILLES is the largest study investigating the imaging characteristics of heel enthesitis in SpA patients to date. Blinded central reading based on HEMRIS confirmed inflammatory and/or structural changes in the majority of the trial population. However, inflammatory changes were not identified for 24% of patients, while MRIs for 17% of subjects were assessed as negative for any enthesopathy-related parameter.

In a previous comparative study of MRI and PDUS in SpA patients and controls, painful heels were found to have more inflammatory abnormalities compared to heels with no pain. However, inflammatory abnormalities were not found in the MRIs of 19% of painful heels, and MRI pathologies of enthesitis were noted in 56% of SpA patients without heel pain or history of heel pain; neither MRI nor PDUS could discriminate between SpA patients and controls [8]. Although the current results further highlight the challenges to understanding the consistency between clinical and imaging assessments of enthesitis, the discrepancy between local assessment and central reading might be partly due to a number of reasons. The clinical assessment of enthesitis might have influenced the evaluation of MRIs by the investigator while the central readers were blinded for any clinical information. Clinical scores might overestimate enthesitis due to confounders such as mechanical stress and fibromyalgia. Additionally, other pathologies found in the heel (proximal mechanical tendinitis, osteoarthritis, cysts) might have been misinterpreted as inflammatory enthesitis. Also, image quality may account for false-positive images as assessed by local investigators; however, a vast majority of all MRIs taken at screening were rated “optimal” by both readers (data not shown). Despite the confounding variables mentioned above, imaging (MRI) is a useful tool to confirm enthesitis, provided there is sufficient experience and expertise of the local radiologist and/or rheumatologist.

Qualitative assessment of the images revealed that most of the pathologies were found in the area of Achilles tendon while the plantar fascia was affected to a much lesser extent. For a substantial number of patients, none or only one inflammatory/structural pathology was identified on MRI. Bursitis, intra-tendon hypersignal, peri-tendon hypersignal, and tendon thickening were scored with higher mean scores and were the most pronounced pathologies. A previous study investigating 27 SpA patients (meeting European Spondyloarthropathy Study Group criteria) with low- and high-field MRI also reported retrocalcaneal bursitis as the most common finding, albeit with a higher proportion in the overall cohort (80%) [18].

At screening, a notable number of patients were found negative for any of the HEMRIS parameters in the area of the Achilles tendon and plantar aponeurosis. In the course of the treatment up to week 24, only very few reported newly developed pathologies when analyzed.

When analyzing the overall population, changes from screening to week 24 of total entheseal inflammation score were mainly observed in the area of the Achilles tendon. The mean and specific inflammatory parameter changes were higher in the secukinumab group compared to placebo most notably for bursitis. In the area of plantar fascia, the mean change in inflammatory score from screening to week 24 was very low. This might be attributed to limited room for improvement as the inflammatory score at screening was less than one on a score range of 0 to 9. In the HEEL trial, a randomized placebo-controlled study in refractory heel enthesitis in patients with SpA, although etanercept has shown significant improvements in patient’s global assessment and heel pain, no significant changes were observed in the MRI when looking into calcaneum bone edema either located at Achilles tendon or in the area of fascia plantaris [19].

In terms of structural abnormalities, minimal changes were observed over time in both the area of the Achilles tendon and plantar fascia, potentially due to the few structural findings at screening, hence the limited room for improvement. In addition, structural parameters usually result from persistent and ongoing disease and are therefore unlikely to improve spontaneously. Interestingly, another prospective study found that chronic, structural changes at the Achilles´ tendon, as assessed by both PDUS and MRI, were more specific than inflammatory changes in distinguishing the peripheral involvement in SpA as compared to non-SpA controls [20]. A slight improvement of placebo patients after switching to active secukinumab treatment at week 24 in both areas of the heel was seen at week 52 for the total entheseal inflammation score and the total structural damage score; however, the increased proportion of missing data through week 52 should be taken into account.

Another interesting question was whether the improvements in imaging parameters are associated to improvements in clinical parameters. Interestingly, an association was only found for peri-tendon hypersignal and heel enthesopathy and for retrocalcaneal bursitis and heel pain. A recent post hoc pooled analysis of 3 previous studies investigated the relationship between physical examination and US, where physical examination of the plantar aponeurosis was uncoupled from the US abnormalities and more abnormalities on US (hypo-echogenicity [*P*<0.001], thickening [*P*=0.01], Doppler signals [*P*=0.002], and erosions [*P*=0.02]) were identified in patients with clinical Achilles tendon enthesitis [18]. In another evaluation with whole-body MRI, Achilles enthesitis was frequently present on MRI but often without clinical signs [21].

ACHILLES is a patient-centered trial addressing the disease burden of heel enthesitis across the spectrum of spondyloarthropathies and applies for the first time before and after treatment with a biologic, a scoring system recently developed by OMERACT to quantify lesions in the heel of SpA patients. However, as this is a post hoc analysis of re-evaluated images from a prospective trial, it was not tailored to the HEMRIS parameters, but rather to the modified PsAMRIS score. This could have impacted the evaluation of a possible association of clinical and imaging parameters. Furthermore, there was no contrast agent applied in ACHILLES while HEMRIS proposes T1-weighted post-gadolinium sequences for entheseal soft tissue inflammation. However, T2-weighted fat-suppressed sequences were available for all ACHILLES subjects, as defined by HEMRIS for evaluation of inflammatory parameter.

## Conclusions

In conclusion, in the ACHILLES trial, 84% of patients presented with entheseal inflammation and/or structural damage considering both the Achilles tendon and plantar fascia. Inflammatory and structural changes were more frequently observed in the area of the Achilles tendon; total entheseal inflammation and structural damage scores were higher to plantar fascia. At week 24, a positive trend in total inflammation and total structural damage scores was observed for secukinumab treated patients compared to placebo with retrocalcaneal bursitis, peri-tendon hypersignal, and BME showing the highest mean changes. The use of HEMRIS to reassess the imaging abnormalities in a cohort of SpA patients provided information on the inflammatory and structural parameters of heel enthesitis and further deepened our understanding of imaging characteristics of clinically assessed enthesitis.

## Supplementary Information

**Additional file 1: Supplemental Figure 1**. Change from screening to week 24 of total entheseal inflammation score and total structural damage score in the area of the Achilles tendon and plantar fascia divided by PsA and axSpA patients. A. Subgroup of patients with underlying indication PsA. B. Subgroup of patients with underlying indication axSpA. axSpA, axial spondyloarthritis; n, number of patients; PBO, placebo; PsA, psoriatic arthritis; SCR, screening; SD, standard deviation; SEC, secukinumab.

**Additional file 2: Supplementary Table 1**. Number of patients with newly developed pathologies from screening to Week 24

**Additional file 3: Supplementary Table 2**. Change from screening to week 24/week 52 of individual MRI parameter: Mean change of HEMRIS scores (overall population). A. Area of Achilles tendon. B. Area of plantar aponeurosis

## Data Availability

The data sets generated during and/or analyzed at the end of the current study are not publicly available. Novartis is committed to sharing with qualified external researchers’ access to patient-level data and supporting clinical documents from eligible studies. These requests are reviewed and approved based on scientific merit. All data provided are anonymized to respect the privacy of patients who have participated in the trial in line with applicable laws and regulations. The data may be requested from the corresponding author of the manuscript.

## Abbreviations

axSpA: Axial spondyloarthritis
BASDAI: Bath Ankylosing Spondylitis Disease Activity Index
BME: Bone marrow edema
CASPAR: ClASsification for Psoriatic ARthritis
HEMRIS: Heel Enthesitis MRI Scoring system
LEI: Leeds Enthesitis Index
MASES: Maastricht Ankylosing Spondylitis Enthesitis Score
MRI: Magnetic resonance imaging
OMERACT: Outcome Measures in Rheumatology
PDUS: Power Doppler ultrasound
PsA: Psoriatic arthritis
PsAMRIS: Psoriatic arthritis magnetic resonance image scoring system
s.c.: Subcutaneous
SD: Standard deviation
SpA: Spondyloarthritis
SPARCC: Spondyloarthritis Research Consortium of Canada
STIR: Short inversion time inversion-recovery
US: Ultrasound
