# Supplementary material for: Magnetic resonance imaging characteristics in patients with spondyloarthritis and clinical diagnosis of heel enthesitis: post hoc analysis from the phase 3 ACHILLES trial
Source: Arthritis Res Ther. 2022 May 16;24:111. doi: 10.1186/s13075-022-02797-8 (PMC9109380; doi:10.1186/s13075-022-02797-8)
Supplement: Supplementary file 1 — Additional file 1: Supplemental Figure 1. Change from screening to week 24 of total entheseal inflammation score and total structural damage score in the area of the Achilles tendon and plantar fascia divided by PsA and axSpA patients. A. Subgroup of patients with underlying indication PsA. B. Subgroup of patients with underlying indication axSpA. axSpA, axial spondyloarthritis; n, number of patients; PBO, placebo; PsA, psoriatic arthritis; SCR, screening; SD, standard deviation; SEC, secukinumab. [file 13075_2022_2797_MOESM1_ESM.pdf]

Supplemental Figure 1. Change from screening to week 24 of total entheseal inflammation score and total structural damage score in the area of the Achilles tendon and plantar fascia divided by PsA and axSpA patients

A. Subgroup of patients with underlying indication PsA

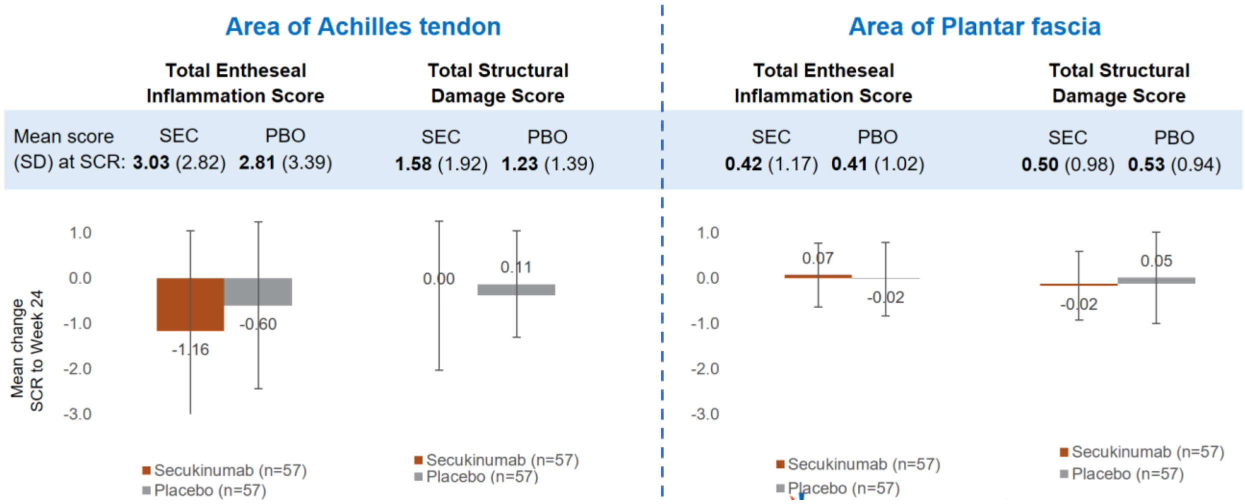

B. Subgroup of patients with underlying indication axSpA

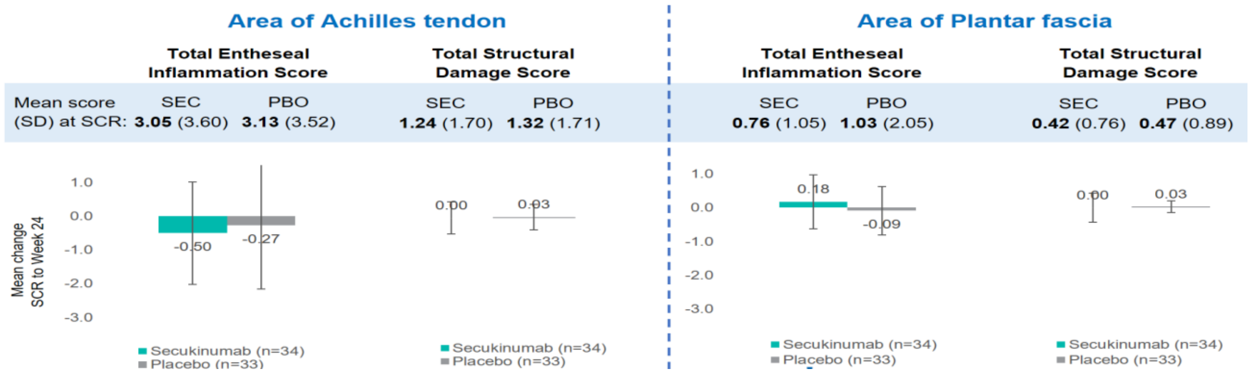

axSpA, axial spondyloarthritis; n, number of patients; PBO, placebo; PsA, psoriatic arthritis; SCR, screening; SD, standard deviation; SEC, secukinumab
