# Supplementary material for: Magnetic resonance imaging characteristics in patients with spondyloarthritis and clinical diagnosis of heel enthesitis: post hoc analysis from the phase 3 ACHILLES trial
Source: Arthritis Res Ther. 2022 May 16;24:111. doi: 10.1186/s13075-022-02797-8 (PMC9109380; doi:10.1186/s13075-022-02797-8)
Supplement: Supplementary file 2 — Additional file 2: Supplementary Table 1. Number of patients with newly developed pathologies from screening to Week 24 [file 13075_2022_2797_MOESM2_ESM.pdf]

Supplemental Table 1. Number of patients with newly developed pathologies from screening to Week 24

MRI Parameter, n/N	SEC	PBO
**Area of Achilles tendon**		
Intra-tendon hypersignal	3/54	0/53
Peri-tendon hypersignal	3/62	2/63
Retrocalcaneal bursitis	1/58	7/60
Bone marrow oedema	4/67	3/74
Tendon thickening	2/66	3/64
Bone spur	2/76	1/82
Bone erosion	2/75	4/84
**Area of Plantar fascia**		
Intra-aponeurosis hypersignal	3/78	0/80
Peri-aponeurosis hypersignal	1/76	1/76
Bone marrow oedema	2/75	2/77
Aponeurosis thickening	0/79	2/76
Bone spur	1/70	2/70
Bone erosion	1/88	1/86

MRI, magnetic resonance imaging; N, total number of patients in the treatment group without respective pathology at screening; n, number of patients with newly developed pathology from screening to Week 24; PBO, placebo; SEC, secukinumab
