# Supplementary material for: Magnetic resonance imaging characteristics in patients with spondyloarthritis and clinical diagnosis of heel enthesitis: post hoc analysis from the phase 3 ACHILLES trial
Source: Arthritis Res Ther. 2022 May 16;24:111. doi: 10.1186/s13075-022-02797-8 (PMC9109380; doi:10.1186/s13075-022-02797-8)
Supplement: Supplementary file 3 — Additional file 3: Supplementary Table 2. Change from screening to week 24/week 52 of individual MRI parameter: Mean change of HEMRIS scores (overall population). A. Area of Achilles tendon. B. Area of plantar aponeurosis [file 13075_2022_2797_MOESM3_ESM.pdf]

Supplementary Table 2. Change from screening to week 24/week 52 of individual MRI parameter: Mean change of HEMRIS scores (overall population)

A. Area of Achilles tendon

**Mean Change (SD) from SCR**		**SEC**	**PBO/SEC**	**95% CI; *P* value**
Intra-tendon hypersignal	Week 24	−0.10 (0.47)	−0.10 (0.60)	(−0.15, 0.15); 0.985
	Week 52	−0.11 (0.53)	−0.10 (0.66)	–
Peri-tendon hypersignal	Week 24	−0.27 (0.92)	−0.11 (0.66)	(−0.36, 0.04); 0.121
	Week 52	−0.23 (0.90)	−0.21 (0.66)	–
Retrocalcaneal bursitis	Week 24	−0.35 (0.79)	−0.08 (0.62)	(−0.42, −0.06); 0.008
	Week 52	−0.34 (0.91)	−0.23 (0.78)	–
BME	Week 24	−0.19 (0.77)	−0.19 (0.86)	(−0.13, 0.23); 0.616
	Week 52	−0.15 (0.66)	−0.26 (0.70)	–
Tendon thickening	Week 24	0.00 (0.39)	0.01 (0.32)	(−0.12, 0.10); 0.830
	Week 52	−0.06 (0.40)	−0.01 (0.60)	–
Bone erosion	Week 24	−0.03 (0.35)	0.07 (0.39)	(−0.19, 0.03); 0.166
	Week 52	−0.01 (0.25)	0.06 (0.34)	–
Bone spur	Week 24	0.03 (0.23)	0.00 (0.15)	(−0.02, 0.09); 0.241
	Week 52	0.01 (0.19)	0.00 (0.00)	–

Data presented as observed. As PBO patients switched to SEC at Week 24, 95% CI and *P* values are not available at Week 52
SEC n-counts at Week 24=91, PBO n-counts at Week 24=90, Week 52=70
CI, confidence interval; n, number of patients; PBO, placebo; SCR, screening; SD, standard deviation; SEC, secukinumab

B. Area of plantar aponeurosis

Mean Change (SD) from SCR		SEC	PBO/SEC	95% CI; *P* value
Intra-aponeurosis hypersignal	Week 24	−0.02 (0.33)	−0.10 (0.50)	(−0.05, 0.14); 0.321
	Week 52	−0.10 (0.34)	−0.10 (0.54)	–
Peri-aponeurosis hypersignal	Week 24	−0.04 (0.25)	−0.08 (0.37)	(−0.06, 0.09); 0.652
	Week 52	−0.09 (0.28)	−0.09 (0.47)	–
BME	Week 24	−0.08 (0.54)	−0.09 (0.71)	(−0.11, 0.17); 0.656
	Week 52	−0.25 (0.74)	−0.19 (0.82)	–
Aponeurosis thickening	Week 24	−0.01 (0.18)	0.01 (0.24)	(−0.09, 0.03); 0.409
	Week 52	−0.03 (0.22)	0.01 (0.21)	–
Bone erosion	Week 24	0.01 (0.10)	0.01 (0.11)	(−0.03, 0.03); 0.904
	Week 52	−0.01 (0.11)	0.00 (0.00)	–
Bone spur	Week 24	−0.01 (0.24)	0.02 (0.15)	(−0.09, 0.02); 0.255
	Week 52	0.01 (0.19)	0.00 (0.17)	–

Data presented as observed. As PBO patients switched to SEC at Week 24, 95% CI and *P* values are not available at Week 52
SEC n counts at Week 24=91, PBO n-counts at Week 24=90, Week 52=70

BME, bone marrow edema; CI, confidence interval; MRI, magnetic resonance imaging; PBO, placebo; SCR, screening; SD, standard deviation; SEC, secukinumab
